# Design of a targeted blood transcriptional panel for monitoring immunological changes accompanying pregnancy

**DOI:** 10.3389/fimmu.2024.1319949

**Published:** 2024-01-30

**Authors:** Tobias Brummaier, Darawan Rinchai, Mohammed Toufiq, Mohammed Y. Karim, Tanwir Habib, Jürg Utzinger, Daniel H. Paris, Rose McGready, Alexandra K. Marr, Tomoshige Kino, Annalisa Terranegra, Souhaila Al Khodor, Damien Chaussabel, Basirudeen Syed Ahamed Kabeer

**Affiliations:** ^1^ Shoklo Malaria Research Unit, Mahidol-Oxford Tropical Medicine Research Unit, Faculty of Tropical Medicine, Mahidol University, Mae Sot, Thailand; ^2^ Swiss Tropical and Public Health Institute, Allschwil, Switzerland; ^3^ University of Basel, Basel, Switzerland; ^4^ Research Department, Sidra Medicine, Doha, Qatar; ^5^ Department of Infectious Diseases, St. Jude Children Research Hospital, Memphis, TN, United States; ^6^ Bioinformatics Core, Weill Cornell Medicine-Qatar, Education City, Doha, Qatar; ^7^ Centre for Tropical Medicine and Global Health, Nuffield Department of Medicine, University of Oxford, Oxford, United Kingdom; ^8^ Computational Sciences Department, The Jackson Laboratory, Farmington, CT, United States

**Keywords:** pregnancy-associated complications, whole blood gene expression, BloodGen3 module, immunomodulatory processes, transcriptome fingerprinting assay

## Abstract

**Background:**

Immunomodulatory processes exert steering functions throughout pregnancy. Detecting diversions from this physiologic immune clock may help identify pregnant women at risk for pregnancy-associated complications. We present results from a data-driven selection process to develop a targeted panel of mRNAs that may prove effective in detecting pregnancies diverting from the norm.

**Methods:**

Based on a *de novo* dataset from a resource-constrained setting and a dataset from a resource-rich area readily available in the public domain, whole blood gene expression profiles of uneventful pregnancies were captured at multiple time points during pregnancy. BloodGen3, a fixed blood transcriptional module repertoire, was employed to analyze and visualize gene expression patterns in the two datasets. Differentially expressed genes were identified by comparing their abundance to non-pregnant postpartum controls. The selection process for a targeted gene panel considered (i) transcript abundance in whole blood; (ii) degree of correlation with the BloodGen3 module; and (iii) pregnancy biology.

**Results:**

We identified 176 transcripts that were complemented with eight housekeeping genes. Changes in transcript abundance were seen in the early stages of pregnancy and similar patterns were observed in both datasets. Functional gene annotation suggested significant changes in the lymphoid, prostaglandin and inflammation-associated compartments, when compared to the postpartum controls.

**Conclusion:**

The gene panel presented here holds promise for the development of predictive, targeted, transcriptional profiling assays. Such assays might become useful for monitoring of pregnant women, specifically to detect potential adverse events early. Prospective validation of this targeted assay, in-depth investigation of functional annotations of differentially expressed genes, and assessment of common pregnancy-associated complications with the aim to identify these early in pregnancy to improve pregnancy outcomes are the next steps.

## Introduction

There is a need for development of new assays and modalities to improve our understanding of immune trajectories during pregnancy. Indeed, pregnancy is a formative experience for expecting mothers and its progression is thought to shape the health and development of the fetus. Research in the emerging field of developmental origins of health and disease (DOHaD) indicates that there might be long-term consequences of pregnancy that affect the lifespan beyond birth and the immediate postpartum period (1). The role of the immune system affecting this process is increasingly recognized. Pregnancy constitutes an evolutionary challenge for the immune system, as there is a constant trade-off in balancing the need for tolerance of the fetal allograft and maintaining adequate protection against environmental stressors (e.g., infections) (2). From conception to birth, a plethora of physiological and immunological changes are required to ensure a successful pregnancy (3). Recent evidence suggests that immune responses play an essential role in all pregnancy stages, from implantation to initiation of labor. A network of different *immune pacemakers* that regulate and time the immune responses during pregnancy, was termed “the human immune clock” of pregnancy by Aghaeepour et al. (3, 4). A disruption of this immune clock may lead to potentially severe pregnancy complications, including fetal loss, preeclampsia, and preterm birth (5–7).

Various omics profiles (e.g., transcriptomics, epigenomics, metabolomics, and microbiomics) exhibit significant temporal changes during pregnancy. Recently the potential of machine learning in predicting gestational age has been demonstrated, with high degrees of correlation between actual and predicted weeks of gestation being reported. These findings underscore the importance of dynamic changes in maternal physiology over the course of pregnancy and the potential utility of omic profiles in understanding these changes and harnessing them for diagnostic and predictive purposes (8–10).

Tarca et al. reported that whole blood gene expression can accurately predict gestational ages in both uneventful and complicated pregnancies (11). Blood samples collected during pregnancy were also able to predict time to delivery in uneventful pregnancies and those with spontaneous preterm birth, a critical finding when considering the utility of omics in preterm birth prediction. Another study identified specific genes (i.a., PAPPA2 and FABP1) as predictors in the gestational age model. This study highlights the imbalance of cell-free RNA signatures between pregnancy progression and pathology, offering insights into how RNA profiles can be used to monitor and understand different stages of pregnancy (12). Liang et al., developed a metabolic clock using five metabolites that accurately predicted gestational age. The study further identified metabolites that could predict the time to delivery within specific time frames, representing a significant advancement in the weekly characterization of human pregnancy using omics approaches (13).

In this context, we aimed to develop a targeted panel of blood transcripts to enable immune monitoring at high temporal resolution in large cohorts of pregnant women for a wide range of study settings. Profiling transcript abundance in bulk blood samples is relatively straightforward and as such is amenable to clinical translation on the following grounds. First, it does not require sample fractionation. Second, profiles can be generated using small volumes of whole blood collected using minimally invasive techniques [e.g., finger sticks (14)]. Third, it permits self-collection by the study subjects, and hence, enables implementation of high frequency sampling protocols (15). As shown in recent studies, immune monitoring at high temporal frequencies contributes to higher resolution of different immune response components, which allows precise mapping of inter-individual differences among study participants (16). Hence, when carried out prospectively, this approach may be suitable for a pre-symptomatic detection of pregnancy associated comorbidities (“disease interception”) (17). Against this background, we designed and implemented the molecular signature in pregnancy (MSP) study, a prospective, longitudinal cohort designed to investigate cross-omic trajectories throughout pregnancy, at delivery and postpartum (18, 19).

While blood transcriptome profiling by RNAseq has become relatively cost-effective, the bioinformatics “overhead” (e.g., infrastructure and expertise) remains high, especially when undertaken on large scales. In our MSP study, for instance, the study protocol required collection of 45 samples from each of the 400 mother and child pairs enrolled, corresponding to more than 16,000 samples. A sensible approach in such circumstances would consist in developing *ad hoc*/fit-for-purpose targeted transcript panels. Typically, a targeted panel comprises tens or hundreds of transcripts, selected from transcriptome profiling data. Cost-effective and scalable technology platforms that require only a limited amount of sample and data processing can be employed for the measurement of transcript abundance for such a targeted panel of genes. Assays implemented on these platforms are also more readily transferrable to resource constrained settings, which is an objective of the MSP study. Lastly, such a downscaling process is necessary when working towards clinical implementation and diagnostic applications.

This paper describes the steps undertaken to inform the development of a targeted blood transcriptional assay for monitoring immune trajectories during pregnancy. We identified gene signatures relevant to pregnancy progression that also reflect inter-individual as well as temporal intra-individual differences in blood transcript abundance in healthy pregnant women. First, we applied transcriptome profiling data generated *de novo* for a subset of healthy pregnant women enrolled in the MSP study. Second, we leveraged a public blood transcriptome dataset that included profiles generated at similar time points for healthy pregnant women from a resource rich setting. Third, for the exploratory gene selection process, both datasets were then analyzed by a well-characterised fixed module repertoire as a framework for the downstream gene selection process. The resulting 176-gene panel will serve as the basis for the development of a multiplexed high throughput polymerase chain reaction (PCR) assay.

## Methods

### *De novo*-generated MSP dataset

#### Setting and participants

We generated whole blood gene expression data from a cohort (trial registration number NCT02797327), that was established in cooperation between Shoklo Malaria Research Unit (SMRU; Mae Sot, Thailand), and Sidra Medicine (Doha, Qatar). The main objective of this prospective pregnancy-delivery-postpartum cohort (“MSP cohort”) was to monitor trajectories of various molecular signatures during the course of pregnancy, at delivery and the early postpartum period. A detailed description of the cohort setting, including definition and procedures, has been published (18). Briefly, the cohort was established from September 2016 to May 2019 in SMRU antenatal care clinics located in a low resource setting on the Thailand-Myanmar border. Pregnant women originating from a migrant population were enrolled in the first trimester of their pregnancy, followed throughout pregnancy, at delivery and in the early postpartum period. At the outset, healthy women with an unremarkable medical and obstetric history were prioritized, but they remained in the cohort and continued to follow if they developed pregnancy-associated complications. Gestational age was confirmed by early obstetric ultrasound and identical routine antenatal care (ANC) procedures were provided to all women.

#### Sampling protocol

For the blood transcriptome profiling component of the study, the sampling protocol consisted of two weekly finger-prick capillary sampling. Fifty microliters of capillary blood were mixed with an RNA stabilizing solution (Tempus™, ThermoFisher Scientific; Waltham, MA, USA) at a 1:2 ratio, shaken vigorously to disrupt and lyse the blood cells to release their RNA, which is then precipitated in the Tempus™ solution and protected from degradation by RNAses (14). Samples were stored at -20°C and later transferred internationally to Sidra Medicine on dry ice in styrofoam boxes equipped with temperature loggers.

#### Sample selection and processing

**Figure 1:**
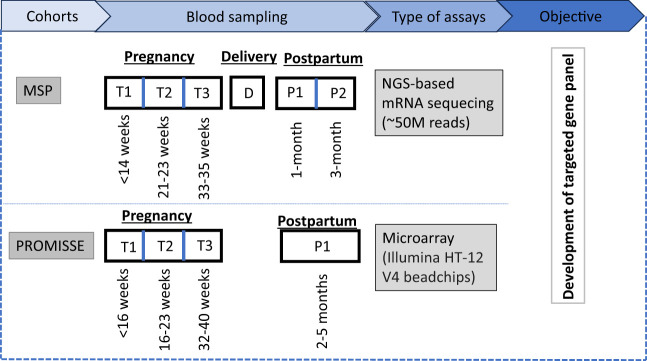
Study overview: The MSP cohort involves blood sampling at three antenatal time points (T1, T2, T3), at delivery (D), and two postpartum time points (P1, P2), analyzed via NGS-based mRNA sequencing with an approximate read count of 50 million. For the PROMISSE cohort, a single postpartum sample (P1) is collected and analyzed using microarray technology, specifically utilizing Illumina HT-12 V4 beadchips. The two transcriptome datasets were used for the development of a targeted gene panel to monitor the pregnancy.

A group of 15 women with an uneventful pregnancy (i.e., normal vaginal delivery at term, absence of common pregnancy-associated complications, such as gestational diabetes, preeclampsia/eclampsia and communicable diseases such as malaria during pregnancy) were selected from the MSP cohort and for each of these whole blood RNA profiles were generated for 6 timepoints: (i) first trimester; (ii) second trimester; (iii) third trimester; (iv) delivery; (v) one month postpartum; and (vi) three months postpartum (**Figure 1**). Demographic and clinical information of study participants is provided in **Supplementary Table 1**.

Total RNA was extracted from the whole blood lysates using Tempus spin Blood RNA extraction kit (Thermo Fisher Scientific Inc., Waltham, MA, USA). Sample integrity and concentration were measured using the standard sensitivity RNA assay on the Perkin Elmer Caliper Labchip GXII (PerkinElmer Inc., Waltham, MA, USA).

#### Sequencing procedures

The Illumina Truseq Stranded mRNA kits (Illumina Inc., San Diego, USA) were used to prepare libraries with 500 ng of total RNA. To obtain mRNA libraries, poly-A RNA selection was performed using an Oligo-dT magnetic bead system, followed by fragmentation, first strand synthesis using Superscript IV and then second strand synthesis. The cDNA obtained after reverse transcription was ligated with IDT for Illumina UD Indexes and amplified for 15 cycles. Library quality and concentrations were assessed using the DNA 1k assay on a Perkin Elmer GX2 (PerkinElmer Inc., Waltham, USA) and quantified using the KAPA HiFi Library quantification kit on a Roche LightCycler 480 (Hoffmann-La Roche AG, Basel, Switzerland). Cluster generation was performed on a cBot 1.0 or 2.0 using the HiSeq^®^ 3000/4000 PE Cluster Kit and HiSeq^®^ 3000/4000 SBS Kit (300 cycles). Flow cells were loaded at a cluster density between 1,310 and 1,524 K/mm^2^ and sequenced to a depth of 20-50 M reads/sample on an Illumina Hiseq 4000 instrument (Illumina Inc., San Diego, USA).

Processing of RNA-Seq data was done using bcbio rnaseq pipeline, bcbio version 1.2.3. Prior to alignment, a quality check of raw data was performed using FastQC version 0.11.9. Alignments were made using STAR version 2.6.1d and reads were mapped to the hg38 genome. After alignment, Samtools 1.3 was used to collect metrics on bam, which were used further to generate a multiqc report.

### Publicly available PROMISSE dataset

#### Rationale

We sought to identify transcriptome profiling datasets generated from women with uncomplicated pregnancies obtained in the context of studies conducted in a high-resource setting. Selection criteria included the availability of non-pregnant reference samples, a control group of uneventful pregnancies and sampling at multiple timepoints. Transcriptome data from the PROMISSE study (Predictors of pRegnancy Outcome: bio- Markers In antiphospholipid antibody Syndrome and Systemic lupus Erythematosus) contributed by Hong et al. were identified as most suitable and downloaded from the GEO browser (GSE 108497) (20).

#### Setting and participants

Details of the PROMISSE study have been described by Hong et al. (20). In brief, the PROMISSE cohort followed a multicentre observational study design and included pregnant women with systemic lupus erythematosus (SLE) and healthy pregnant controls. All participants originated from a high-resource setting (i.e. Canada and USA). Blood was drawn at 5 prespecified timepoints: (i) <16 weeks’ gestation (WG); (ii) 16-23 WG; (iii) 24-31 WG; (iv) 32-40 WG; and (v) between 8 and 20 weeks postpartum. PAXgene Blood RNA Kit (Qiagen, Venlo, The Netherlands), a system for collection, stabilization and purification of RNA was used to obtain transcriptome trajectories.

#### Sample selection and processing

Healthy pregnant controls (n=38) were selected from the PROMISSE cohort. Sample processing differed from the *de novo* MSP dataset as after extraction and amplification, RNA was hybridized to Illumina HT-12 V4 beadchips (Illumina Inc., San Diego, USA) that contained 47,231 probes. Once scanned on an Illumina Beadstation 500, signal intensities were generated, background was subtracted, and data were normalized by using Illumina’s GenomeStudio. This microarray dataset was then downloaded from the GEO browser and gene expression data were extracted for downstream analysis.

### Data analysis and visualization

#### BloodGen3 repertoire

Analysis for both datasets was carried out using the BloodGen3 fixed blood transcriptional module repertoire, for which detailed information is published (21). Briefly, the approach is based on the delineation of co-expressed sets of genes (“modules”) for a given system (e.g., the whole blood transcriptome). The BloodGen3 module repertoire was constructed based on 16 distinct datasets spanning over different pathological and physiological conditions, including a wide range of autoimmune and infectious diseases, as well as transplantation, cancer and pregnancy. It is based on a total of 985 individual transcriptome profiles. Dimensions were reduced to 382 sets of co-expressed genes, which were identified via construction and mining of a weighted co-clustering network. In a second step, k-means clustering was employed to further reduce dimensions and define a set of 38 “module aggregates”. The module repertoire is fixed in that it is re-used for analysis and interpretation of datasets that were not employed for its construction. An extensive functional annotation and interpretation framework for the BloodGen3 repertoire was established based on, for instance, gene ontology, pathway or literature term enrichment and transcript profiles. An R package was developed to support module-level data analysis and visualization employing the BloodGen3 repertoire (22). Finally, web applications associated with recent publications provide access to analyzed results for different blood transcriptome dataset collections (for 16 reference cohorts (21), and 6 respiratory syncytial virus (RSV) infections (23). Overall, the use of this fixed repertoire helped streamline the analysis and visualization of blood transcriptional data and to contextualize its interpretation (24).

#### Data analysis

The code used for module repertoire analysis and visualization is available as an open source R package (i.e., *BloodGen3Module* Package) at https://github.com/Drinchai/BloodGen3Module or https://bioconductor.org/packages/release/bioc/html/BloodGen3Module.html and is described in detail elsewhere (22). The workflow consists in the following steps. First, annotating the expression matrix (DESeq2 normalized counts in the case of the MSP dataset, background subtracted and quantile normalized expression values in the case of the PROMISSE dataset) with module repertoire information (mapping transcripts to BloodGen3 modules). Second, identifying differentially expressed genes, which can be done at either the group-level or individual sample-level. P-value and false discovery rate cut-offs were applied (DESeq2 FDR <0.1). When determining changes at the individual sample level the module response is determined by employing fixed fold-change and expression difference cut-offs (|FC|>1.5 and |DIFF|>10). Third, assessing the “module response” that is defined as the percentage of constitutive genes found to be differentially expressed between two study groups, or for the same individual in comparison to a given baseline. The values, therefore, can range from +100% (all constitutive transcripts increased) to −100% (all constitutive transcripts decreased). The dominant trend (i.e., increase or decrease in abundance over control/baseline) was retained for visualization purposes.

For our study, the average absolute, normalized expression values of all 3-month postpartum samples were calculated and used as reference for each analyzed gene in the MSP dataset. An identical approach was chosen for the PROMISSE dataset using the respective postpartum samples. To determine whether a gene was significantly up- or downregulated, levels of gene abundance at different sampling timepoints were compared against the mean expression at the postpartum reference timepoint using *t*-test. A fold-change of at least 1.5 and a *p*-value of less than 0.1 was determined as cut-off for significance.

#### Data visualization

**Figure 2:**
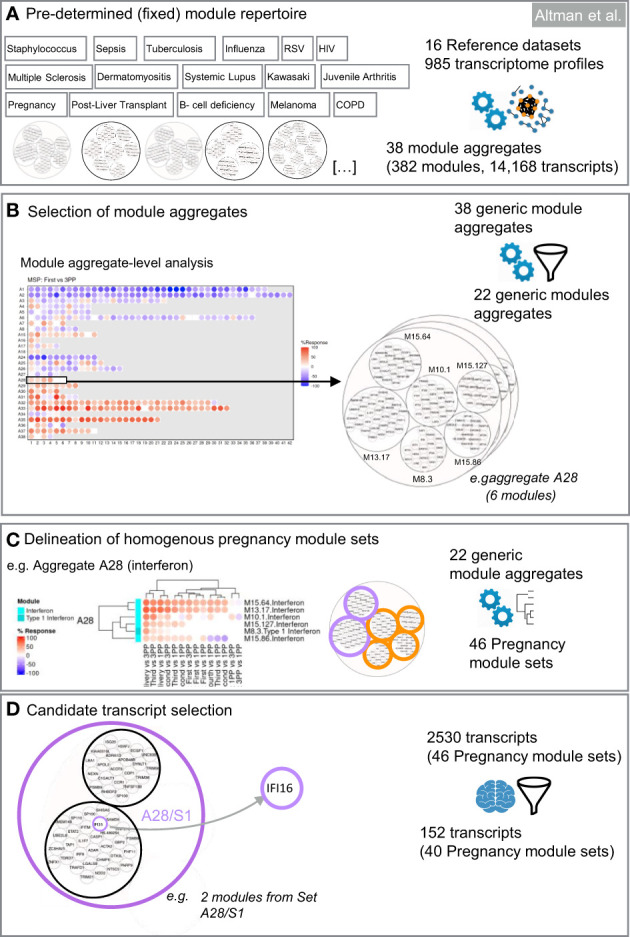
Design of targeted blood transcript panels for pregnancy profiling. The first selection steps are data-driven **(A–C)**. They consist in identifying co-expressed sets of transcripts to constitute selection pools. The last selection step is knowledge-driven **(D)**. It consists in identifying transcripts among each of the pools which are relevant functionally for pregnancy based on a review of the literature. **(A)** Pre-determined module repertoire. The process primarily relies on a generic collection of co-expressed gene sets (transcriptional modules). Two dimension reduction levels are built into this modular repertoire. The most reduced level has 38 variables (module aggregates). The least reduced level has 382 variables (modules). **(B)** Selection of module aggregates. Analysis of expression trajectories within the module aggregates from the first trimester to postpartum was the foundation for selecting 22 of the 38 module aggregates in the first step of dimension reduction. **(C)** Delineation of homogeneous pregnancy module sets. The next step identifies within each of the 40 aggregates subsets of modules that show high degree of expression similarity across pregnancy, at delivery and the postpartum period. **(D)** Candidate transcript selection. The last steps involves expert curation and consists in identifying at least one transcript within each module set. Criteria for selection can be adapted based on needs (e.g. enrichment in candidates that are immune relevant and/or relevant to pregnancy biology).

To enable visual interpretation of the transcriptome fingerprints, modules were arranged on a 2-dimensional grid and assigned to a fixed position. Direction of gene expression compared to the postpartum reference sample was represented by a binary color code: upregulated modules are depicted in red, downregulated modules in blue (**Figure 2**). A variable degree of color saturation illustrates the magnitude of the difference in expression. The level of saturation is dependent on the percentage of up- or downregulated genes within one module. For instance, module M13.26 consists of 40 genes. If all 40 genes (= 100%) of module M13.26 are upregulated, a red spot with the highest saturation is depicted. If no gene in a given module is up- or downregulated (= 0%), the respective spot on the heatmap is blank. The modules were then further grouped into “aggregates” via a second round of clustering, resulting in a lower level of granularity and thus permitting a refined level of interpretation of observed gene expression patterns. Perturbations of transcript signatures in module aggregates follow the same color coding as laid out above.

#### Web application

The R package *Shiny* was used to build an interactive tool (available at: https://immunology-research.shinyapps.io/BloodGen3_Pregnancy) to explore, compare and visualize gene expression patterns from both datasets (25). Additional information describing the composition of aggregates and modules, gene ontologies, expression profiles, functional annotation, among others, are accessible via links to interactive presentations created by the visual communication software Prezi (Budapest, Hungary).

#### Gene selection process

For the selection of a targeted panel, genes represented in the 382 modules forming the BloodGen3 repertoire were condensed through a data- and knowledge-driven selection process that combined test statistics and a review of the literature to identify genes that have been shown to play a role in pregnancy biology. Selection is first conducted at the module aggregate-level. Next, within each of the selected aggregates, modules that show a high degree of collinearity in pregnancy were grouped in “module sets” (i.e., each aggregate could encompass multiple sets, but those do not cross over to other aggregates). Genes are then selected to represent each of these sets, which permits to capture the breadth of the changes in transcript composition observed during the course of pregnancy.

#### Module aggregate selection

The first filter removed module aggregates showing no or only modest changes throughout the course of pregnancy. If the average response of all the modules constituting an aggregate was <10%, this aggregate was not retained in the downstream selection process. As described above, the % response is defined as the proportion of transcripts for a given module up- or downregulated in comparison to the post-partum baseline.

#### Module set selection

The next step was to select only modules showing the highest level of changes throughout pregnancy (i.e., modules with an absolute response of >10%). If >10 modules met this criterion, only the top 10 modules were selected.

Next, we identified homogenous module sets exhibiting coherent expression patterns. Within the selected aggregates, we aimed to identify homogenous module sets exhibiting coherent expression patterns. The top 10 modules within each aggregate, ranked by average response, underwent hierarchical clustering for both the MSP and PROMISSE cohorts independently. Consistent co-clustering across both cohorts was the primary criterion for defining a module set, ensuring that the identified patterns were not cohort-specific artifacts but rather reflected underlying biological processes occurring across diverse populations. This approach has previously been tested and is reported elsewhere (26).

Modules within an aggregate that consistently clustered together in both cohorts were designated as a set. For instance, in the case of aggregate A28, the largest cluster of co-clustering modules in both cohorts was defined as set 1 (A28/S1), and subsequent clusters as set 2 (A28/S2), and so on (**Supplementary Figure 1**). To maintain clarity and facilitate interpretability, a maximum of three module sets per aggregate was established. This parameter was guided by the need to ensure that each set represented a distinct biological signal without becoming unwieldy for practical assay development.

In instances where a cluster was observed to form a distinct group within a single cohort but did not align with clusters from the other cohort, it was categorized separately to preserve the integrity of the biological signal it may represent. Non-clustered modules, defined as those not demonstrating a consistent pattern across datasets, were aggregated into a non-co-clustered set, ensuring that these variable patterns were still captured for potential biological insight.

#### Gene set selection

Representative genes were next selected for each of the selected module sets in a staggered 3-step process factoring in (i) transcript abundance in whole blood; (ii) degree of correlation with the module average; and (iii) pregnancy biology. A maximum of four representative genes were allowed for each of the module sets.

*Step GS1*: The first selection step took transcript abundance into account. The median count of each gene was calculated. All genes with a median count <50 were excluded in this step. This selection could operate only for the MSP dataset since the PROMISSE study dataset was generated using microarrays.

*Step GS2:* Second, correlation of the gene expression trajectories of individual genes within a module set was compared to the average expression value of the module set and linear correlation coefficients (*r*) were generated for each gene (**Supplementary Figure 2**). The genes with an “*r*” value <0.5 and/or *p*-value >0.05 in one or both the datasets (i.e., MSP and PROMISSE) were excluded in this step. The average “*r*” value for a given gene in both datasets was considered as its “R score”. For example, if the “*r*” value of Gene A is 0.8 and 0.6 in MSP and PROMISSE datasets respectively, then the “R score” for Gene A is 0.7.

*Step GS3:* Lastly, the relevance to pregnancy biology was assessed for transcripts selected in the previous steps. Literature profiles of individual genes were obtained by searching the PubMed/Medline database using the following search algorithm: “*gene symbol* [tiab] AND [pregnancy (tiab) OR pregnant (tiab) OR gravid (tiab)]”. If the gene name appeared in the manuscript title a “literature score” amounting to 2 points was given to that gene. If the gene name appeared only in the abstract, then the literature score for that gene was 1. For all other genes, the literature score was 0. The sum of literature score and R score determined the final score. Genes with highest final scores in each module set were selected for TFA panel design (**Supplementary Figure 2**).

To obtain the desired number of gene targets for validation experiments (n=176 test and 8 housekeeping (HK) genes), at least one gene was added from each aggregate that was not yet represented in the gene panel based on a similar process of expression magnitude and literature profiling.

To select the 8 HK genes, (i) four genes were included as HK genes in a generic 272 gene panel that we have designed and implemented previously (27); (ii) two genes were selected based on low coefficients of variation (%CV) in the MSP and PROMISSE datasets; and (iii) the last two genes were selected based on earlier publication reports.

### Statistical analysis

The statistical environment R (version, 4.2.2; Vienna, Austria) was used for all test statistics (28). The R package BloodGen3Module was used for module repertoire analysis and visualization (23). The R package *Shiny* was used to build the interactive online tool for visualization and comparison of expression patterns (26).

### Ethics statement

The *de novo* generated dataset (i.e., MSP) was derived from a prospective study that was approved by the ethics committee of the Faculty of Tropical Medicine, Mahidol University, Bangkok, Thailand (reference no. TMEC 15–062, initial approval 1 December 2015), the Oxford Tropical Research Ethics Committee (reference no. OxTREC: 33–15, initial approval 16 December 2015) and reviewed by the local Tak Province Community Ethics Advisory Board (29). The study was conducted in full conformity with the Declaration of Helsinki and followed regulations of the ICH Guidelines for Good Clinical Practice.

The publicly available dataset was derived from the PROMISSE study, which was reviewed and approved by recruiting organizations institutional review committees: Hospital for Special Surgery Institutional Review Board (IRB), Intermountain Health Care Urban Central Region IRB (Utah, USA), NYU School of Medicine IRB, Oklahoma Medical Research Foundation IRB, The Johns Hopkins Medical Institutions (Western IRB), The University of Chicago IRB, Mt. Sinai Hospital’s Research Ethics Committee (Toronto, Canada), and University of Utah IRB (20).

## Results

### Establishing reference blood transcriptome datasets

The data-driven design for the transcriptional panel incorporated the *de novo* MSP study dataset and the publicly available PROMISEE dataset. The MSP study enrolled 430 pregnant women during their first trimester, recruited between September 2016 and July 2018. For 88.6% (381/430) of these women, an outcome was available (19). Fifteen women with uneventful pregnancies were selected for inclusion in this secondary analysis, and 88 RNAseq profiles were generated from samples collected via capillary finger stick sampling at 6 timepoints: first trimester (n=15), second trimester (n=15), third trimester (n=15), delivery (n=15), 1-month postpartum (n=13) and 3-month postpartum (n=15). The resulting MSP dataset was deposited in the NCBI GEO/SRA database (accession number PRJNA898879) and is available for re-use by third parties.

The reference PROMISSE transcriptome dataset was established by Hong et al. and deposited under the ID GSE108497 in the Gene Expression Omnibus (20). Overall, 155 pregnant women were enrolled in the PROMISSE study, of which 43 were healthy controls (i.e., non-SLE). From the four predefined sampling timepoints, the following numbers were available for each timepoint: estimated gestational age (EGA) <16 weeks (n= 38), EGA16-23 weeks (n= 37), EGA 24-31 weeks (n= 37), EGA 32-40 weeks (n= 35) and postpartum (n= 17). A comparison of the basic characteristics of the two cohorts is presented in **Supplementary Table 2**.

Thus we first obtained the reference transcriptome dataset that included both, the *de novo* generated and publicly available dataset, to inform the design of a targeted blood transcriptional profiling assay for pregnancy monitoring.

### Module repertoire analyses and high-level interpretation of the pregnancy transcriptome fingerprint

**Figure 3:**
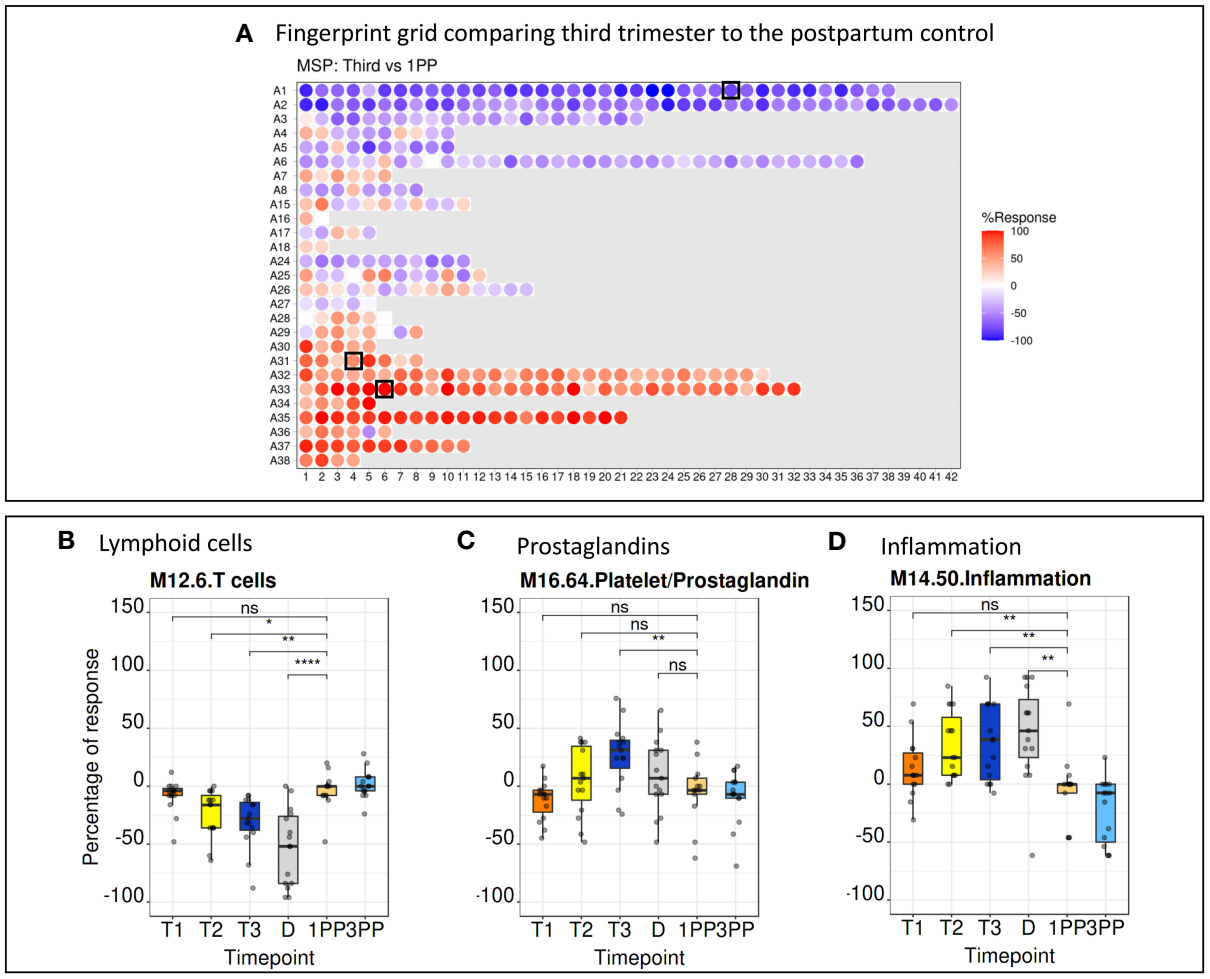
Fingerprint grid plots and functional annotations. **(A)** Fingerprint grid plot comparing the third trimester gene expression pattern to the postpartum control. Red spots showing increase in transcript abundance and blue spot decreases. The position of the modules on the grid is fixed. The black boxes indicate modules discussed in detail below. **(B)** A steady decrease in T-cell function as represented by module M12.6 (position A1.28 on the grid map) is seen throughout pregnancy. **(C)** An increase of prostaglandins from the first to the third trimester is seen in module M16.64 (position A31.4 on the grid map). **(D)** Aggregates associated with inflammation, represented by module M14.50 (position A33.6 on the grid map), show a steady increase from the first trimester to the delivery. 1PP, 1-month postpartum; 3PP, 3-month postpartum; D, delivery; MSP, molecular signature in pregnancy; ns, non-significant; T1, first trimester; T2, second trimester; T3, third trimester. * p<0.1, ** p<0.05, **** p<0.0001.

BloodGen3 module repertoire analyses were carried out using a custom R package (see methods for details) and results made available via an interactive web application (https://immunology-research.shinyapps.io/BloodGen3_Pregnancy/#). Changes in transcript abundance at different time points were mapped against a fingerprint grid where BloodGen3 modules are arranged in rows based on module aggregate membership (the first row corresponding to modules comprised in module aggregate A1, the second row corresponding to modules in aggregate A2, and so on). Fingerprint grid maps for each data point can be accessed via the web application. The pregnancy blood transcriptome fingerprint thus obtained will be the object of more in depth investigations in a follow-up paper. Several observations were made when examining the fingerprint grid map, from a high-level perspective. First, fingerprint grid maps show extensive changes in transcript abundance throughout pregnancy when compared to the postpartum controls (**Figure 3A**). These changes are already evident in the first trimester of pregnancy (**Supplementary Figure 3**) supporting previous reports that highly dynamic immunological changes are present in all stages of pregnancy (2). Second, analogous expression patterns were noticed when comparing data from the low-resource (MSP dataset) to the high-income setting (PROMISSE dataset), suggesting a corresponding immunophysiology in both populations (**Supplementary Figure 3**). Third, changes in aggregates A1-A3 were observed for modules associated with the lymphoid compartment. In aggregate A1, this includes modules M15.33, M12.6, M15.38 and M14.42 that are associated with T-cells and modules M13.27, M15.4, M16.78, M13.18 and M12.8 that are associated with B-cells. These modules exhibited a decrease in transcript abundance compared to the postpartum control. When focusing on a more granular level of the module repertoire, module M12.6 (from aggregate A1) associated with T-cell functions, a steady decline from the first trimester through to delivery was noted, especially in the MSP cohort. The extent of the decrease in transcript abundance of module M12.6 was in the -40% range, which corroborates that the abundance of the transcripts constituting M12.6 is lower when compared to non-pregnant controls (**Figure 3B**). Fourth, gradual increase in expression of module M16.64 (aggregate A31) was noted from the first to the third trimester of pregnancy. This observation is more pronounced in the MSP dataset when compared to the PROMISSE dataset. M16.64 is broadly associated with platelet and prostaglandin activity, whereas prostaglandins play an important role at later stages in pregnancy for cervical ripening and induction of labour (**Figure 3C**) (30). Fifth, robust changes were observed for aggregates A33 and A35, both associated with inflammation. Detailed examination of aggregates A33 and A35 from the MSP dataset, shows that the number of modules in which 100% of the comprising genes (i.e., red color with the highest level of saturation) are upregulated, increases from one in the first trimester (module M14.19) to 8 in the second (modules M13.16, M14.19, M14.26, M14.50, M14.66, M14.76, M14.74 and M15.78) and third (modules M13.16, M14.19, M14.26, M14.50, M14.76, M15.90, M14.74 and M15.78) trimester (**Figure 3D**).

### Selection of module aggregates and identification of coherent sets of pregnancy-relevant modules

**Figure 4:**
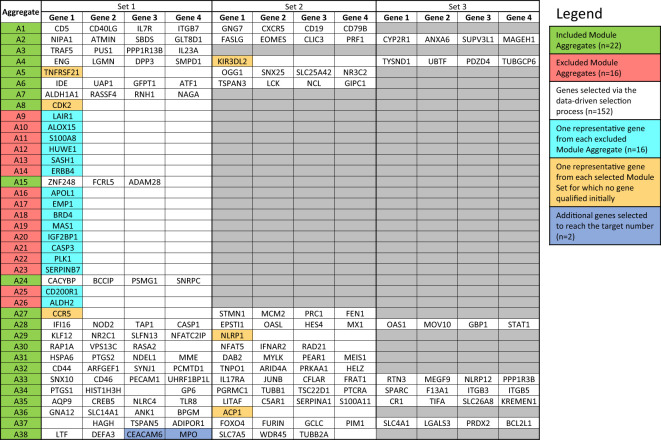
List of genes included in the targeted panel. According to the desired number (i.e., 176 genes and 8 housekeeping genes), target genes were selected following the data-driven selection process described in the Method section. The initially identified 152 genes were complemented by adding (i) one representative gene from each Module Aggregate that was excluded in the first step (n=16), (ii) one gene from the 6 Module Sets for which no gene qualified initially (n=6) and (iii) additional genes from Module Sets for which the maximum number of allowed genes was not reached (n=2).

To select a panel of relevant genes from a pool of thousands of candidate genes, the first filter (see “MA selection”) retained 22 module aggregates, for which at least one constitutive module met a cut-off of 10% for the average response measured across all MSP samples. These aggregates (**Figure 4**) were used for subsequent selection within each aggregate of homogeneous module sets (“MS selection”). Hierarchical clustering was employed to group the top 10 modules (ranked by average % response) for both the MSP and PROMISSE cohorts. Modules from a given aggregate were selected when consistent co-clustering was observed in both cohorts. For instance, as illustrated in **Supplementary Figure 1**, the largest number of modules found to co-cluster in both the MSP and PROMISSE cohorts for aggregate A28 was assigned to set 1 for this aggregate (noted as A28/S1): these are modules M15.65 and M13.17. In turn, one module was assigned to set 2 (A28/S2): M8.3. The rest of the modules that did not display consistent co-clustering patterns were included in a third set (A28/NS: M15.86, M10.1 and M15.127). After the first filter, 46 module sets were retained.

### Selection of genes and design of a targeted panel

**Figure 5:**
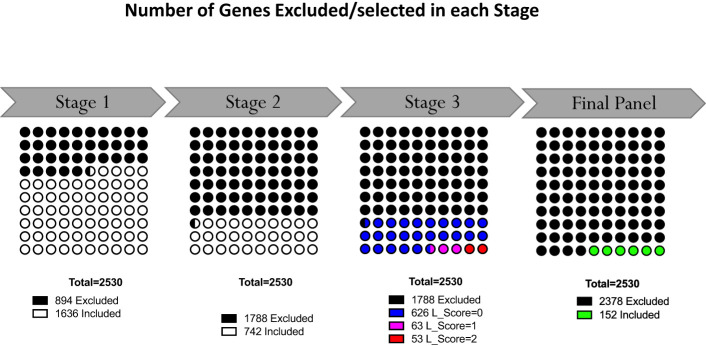
Selection of representative genes. Following Module Aggregate and Module Set delineation, number of genes excluded in the gene selection process at each step. At the outset 2530 genes were contained in the 46 selected Module Sets. Following the staggered 3-step selection process, 152 gene were left.

The last filter at the gene-level (“GS selection”), involved a staggered 3-step screening process, which identified a maximum of four genes per module set. This approach was applied to the pool of 46 pregnancy module sets delineated earlier that encompass 2,530 gene transcripts. Of these, 894 (35.3%) were removed in the first GS1 selection process, as they exhibited a median count smaller than 50. In the second step (GS2), the correlation of each individual gene with the mean of their respective module set was assessed using correlation plots. Correlation analyses compared expression trajectories of selected genes over the course of pregnancy to the average of the module set containing the corresponding gene (**Figure 5**). This step excluded 1,788 genes, which had *r* values <0.5 or *p*-values >0.05. For the remaining 742 genes, literature scores were generated which permitted the prioritization of candidates based on their degree of association with pregnancy in the biomedical literature (GS3: see methods as well as **Supplementary Figure 2** for details). This final knowledge-driven step identified 152 genes, which were then selected after ranking according to literature and correlation scores (i.e., with the GS selection step eliminating 94% of the 2,530 potential candidates). **Figure 5** depicts a graphical summary of the number of genes included/excluded at each step of the gene selection process. The maximum allowable number of four genes per module set was selected from 33 module sets (132 genes), three genes were selected from 6 module sets (18 genes) and two genes from one module set (2 genes), resulting in 152 target genes (**Figure 4**). For 6 module sets, no genes were identified as the defined criteria were not met. Our intent is to implement this assay on the fluidigm BioMark high throughput microfluidics PCR platform. The format of the microfluidics chip used by this instrument supports simultaneous measurement of the abundance for 96 transcripts. Hence, 24 additional targets were selected, along with 8 HK genes to raise the number of transcripts included in this pregnancy blood transcriptome fingerprinting assay to 184. One representative gene was selected for each aggregate excluded in the first step (n=16), one for each module set for which no target gene was identified (n=6) and two genes represented in module set A38/1 were added, i.e., CEACAM6 and MPO (**Figure 4**).

As expected, there was a strong correlation observed between the average expression of all genes within a module and the expression patterns of the selected genes from that module (**Supplementary Figure 4**). The genes selected from aggregates A1, A2, A3, A4, A5, A6, A15, and A29 exhibited a linear downregulation from the first trimester to delivery, followed by a marked upregulation postpartum. Conversely, genes from aggregates A28, A30, A31, A32, A33, A34, A35, A36, A37, and A38 showed an upregulation trend, peaking in the third trimester.

## Discussion

There are an estimated 213 million pregnancies annually worldwide (31). In absolute numbers most pregnancies occur in low- and middle-income countries (LMICs) (32). Various complications can affect pregnancy outcomes and lead to maternal as well as fetal morbidity and mortality. Combined with an often lacking of adequate ANC, pregnant women from LMICs are at higher risk of adverse pregnancy outcomes. Simple and cost-effective tools that enable universal pregnancy monitoring and support detection of potential deviations from the expected norm are needed. Pregnant women with a conspicuous screening result could be kept under closer observation, referred to specialists and/or tertiary hospitals with advanced neonatal care facilities.

The exploratory work presented here aimed to establish a comprehensive panel of whole blood gene transcripts designed to inform about the state of the pregnancy and enable monitoring for potential complications. To determine whether a gene expression signature is altered from the expected physiological pattern, a baseline must be established. This can only be done by examining uneventful pregnancies without pregnancy-associated complications and comparing the genome-wide gene expression patterns to non-pregnant controls. Comparison of physiological pregnancy-associated changes to their own non-pregnant control reduces bias introduced by inter-individual differences in expression patterns. In both datasets employed in this study, pregnant women served as their own, non-pregnant control after successful delivery. Combining datasets from two distinct populations from two different socioeconomic and environmental settings, increased the power of the analysis and broadened interpretation and generalizability of the findings.

Effectively, transcriptome profiling measures the abundance of tens of thousands of transcripts and interpretations down to individual gene levels are strenuous and limit functional interpretation substantially. Hence, selecting panels comprising only hundreds of transcripts from this pool is a challenge. Using selection strategies based on lists of differentially expressed genes tend to bias selection towards the most prominent signatures (with multiple genes from the same signature displaying the highest fold changes and lowest *p*-values). We employed a recently characterized fixed blood transcriptional module repertoire as a framework for the selection of a transcript panel designed for pregnancy monitoring. The 382 modules forming the BloodGen3 repertoire were constructed based on co-expression patterns observed across a collection of 16 reference cohorts, representing a wide range of pathologies and physiological states, including pregnancy (21). The 382 BloodGen3 modules are distributed across 38 “module aggregates”, which are constituted based on co-expression patterns across modules, and hence, providing a second level of dimension reduction (21).

The fixed, reusable blood transcriptional module repertoire BloodGen3 has two advantages. First, it permits reduction of dimensions. Second, it allows inference on biological functions of mRNA products. Moreover, it is a robust tool that is under continuous review and development, and has been used to analyse and interpret gene expression studies in other pathophysiological states (26, 33).

The fixed grid plots allow grasping major differences between different populations or sampling timepoints at a glance. Accordingly, adaptations in the maternal immune system that are required to host the human embryo can be demonstrated and investigated throughout pregnancy. These adjustments in the immune system start at the time when trophoblast cells breach the epithelial lining of the decidua and create an interface between the maternal immune system and fetal antigens (2). The understanding of the underlying immune-physiological changes in pregnancy are still under investigation. However, whole blood gene expression patterns can be considered as unbiased, as no preselection of parameters of interest is performed.

The data-driven selection of whole blood transcript panels for pregnancy monitoring presented here, was based on two blood transcriptome reference datasets from a low-resource (MSP) and high-income setting (PROMISSE) (18, 20). Gene expression profiles of uneventful, term pregnancies were generated at multiple time points during pregnancy as well as postpartum and systematically interpreted for selection of relevant genes. Selecting representative genes may facilitate translation from bench to bedside as a small number of genes could be printed on a chip or a targeted rtPCR battery could be developed, clearing the path for systematic application at lower cost. Testing the robustness of the preliminary selected gene panel is paramount; hence a pragmatic approach led to the determination of the sample size of target genes, which was set to 176 test genes and 8 HK genes. This was based on the fact that the Fluidigm 96.96 Dynamic Array™ test chip (Standard BioTools Inc., San Francisco, USA) will be used to run validation experiments and one chip has 96 wells, 88 test samples and 8 HK genes can be analyzed. Since two chips will be used for each sample in the validation process, there is space for 176 target genes and 8 HK genes (identical HK genes will be used on both plates). It is expected that the number of targets may be reduced following validation experiments. Downsizing of the target panels may prove beneficial for translation into clinical service and therefore increase availability in LMICs, as more widely available real-time PCR systems could be used in the field.

The optimal number of sampling timepoints remains to be determined. The work presented here is based on the analysis of samples taken in each trimester; however, in the overhead MSP cohort sampling frequency was much higher (i.e., 2-weekly). Whether sampling in each trimester or a trade-off between a denser sampling schedule [e.g., at each of the 8 recommended ANC visits by the World Health Organization (WHO) (34)] but less gene targets provides more accurate results remains to be determined.

Lastly, while the main objective of this manuscript was to lay out the selection process for a gene panel for potential pregnancy monitoring, some functional annotations (e.g., downregulation of the lymphoid compartment, steady increase of signatures associated with inflammation) are highlighted. A detailed assessment and interpretation of temporal changes throughout pregnancy and at delivery is in preparation.

The main objective of identifying a panel of genes for potential pregnancy monitoring is based on a gene selection process that has certain methodological limitations. The assumption of co-expression implies positive correlations within gene sets, however, while a common practice, it may not fully capture the complexity of gene regulatory networks. Additionally, our criteria for module aggregate and set selection were influenced by practical constraints.

The integration of quantitative expression data with qualitative literature-based relevance introduces potential biases. The literature scoring system, designed to reflect the gene’s established relevance in pregnancy biology, may inadvertently favor well-characterized genes over less-known yet potentially significant ones. Furthermore, the method of combining literature scores with R scores, intended to balance empirical evidence with established biological knowledge, was not optimized through a formal analytical framework. We recognize that this aspect of the methodology as a limitation that could be refined in future studies.

## Data availability statement

The original contributions presented in the study are publicly available. This data can be found here: PRJNA898879.

## Ethics statement

The studies involving humans were approved by Ethics committee of the Faculty of Tropical Medicine, Mahidol University, Bangkok, Thailand (reference no. TMEC 15–062), The Oxford Tropical Research Ethics Committee (reference no. OxTREC: 33–15) and reviewed by the local Tak Province Community Ethics Advisory Board. The studies were conducted in accordance with the local legislation and institutional requirements. The participants provided their written informed consent to participate in this study.

## Author contributions

TB: Conceptualization, Data curation, Formal analysis, Investigation, Methodology, Validation, Visualization, Writing – original draft, Writing – review & editing. DR: Conceptualization, Data curation, Formal analysis, Investigation, Methodology, Validation, Visualization, Writing – review & editing, Software. MT: Data curation, Formal analysis, Investigation, Methodology, Software, Validation, Visualization, Writing – review & editing. MYK: Formal analysis, Investigation, Writing – review & editing. TH: Formal analysis, Investigation, Writing – review & editing. JU: Writing – review & editing, Project administration, Supervision. DHP: Project administration, Supervision, Writing – review & editing. RM: Project administration, Supervision, Writing – review & editing, Data curation. AKM: Project administration, Supervision, Writing – review & editing. TK: Project administration, Supervision, Writing – review & editing. AT: Project administration, Supervision, Writing – review & editing. SAK: Project administration, Supervision, Writing – review & editing, Funding acquisition. DC: Funding acquisition, Project administration, Writing – review & editing, Conceptualization, Data curation, Formal analysis, Investigation, Methodology, Software, Validation, Visualization, Writing – original draft. BSAK: Conceptualization, Data curation, Formal analysis, Investigation, Methodology, Validation, Visualization, Writing – original draft, Writing – review & editing.
