# Supplementary material for: Design of a targeted blood transcriptional panel for monitoring immunological changes accompanying pregnancy
Source: Front Immunol. 2024 Jan 30;15:1319949. doi: 10.3389/fimmu.2024.1319949 (PMC10861739; doi:10.3389/fimmu.2024.1319949)
Supplement: Supplementary file 1 [file DataSheet_1.zip › Supplement for paper_Final.pptx]

## Slide 1
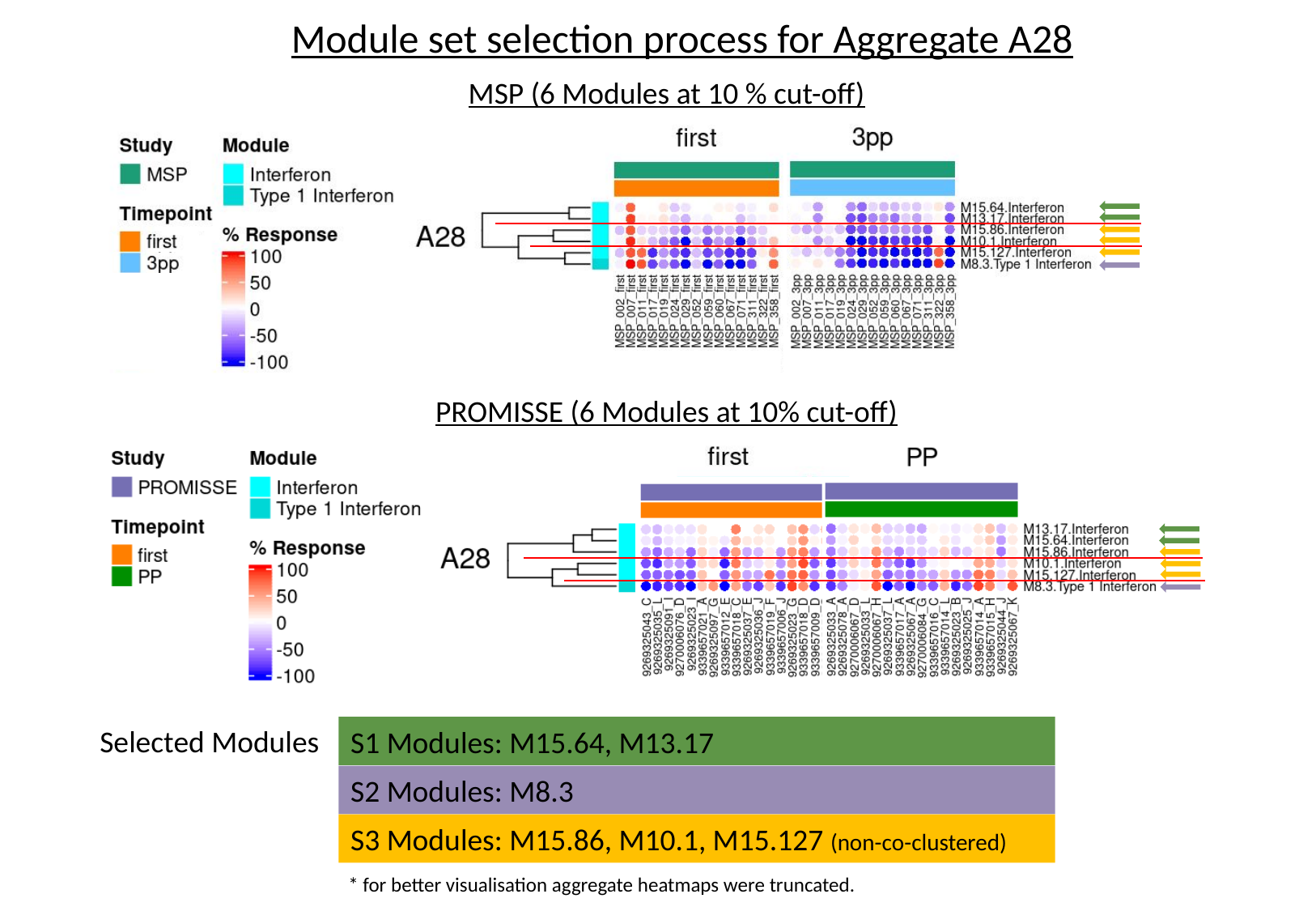

Module set selection process for Aggregate A28
MSP (6 Modules at 10 % cut-off)
PROMISSE (6 Modules at 10% cut-off)
Selected Modules
S1 Modules: M15.64, M13.17
S2 Modules: M8.3
S3 Modules: M15.86, M10.1, M15.127 (non-co-clustered)
* for better visualisation aggregate heatmaps were truncated.

## Slide 2
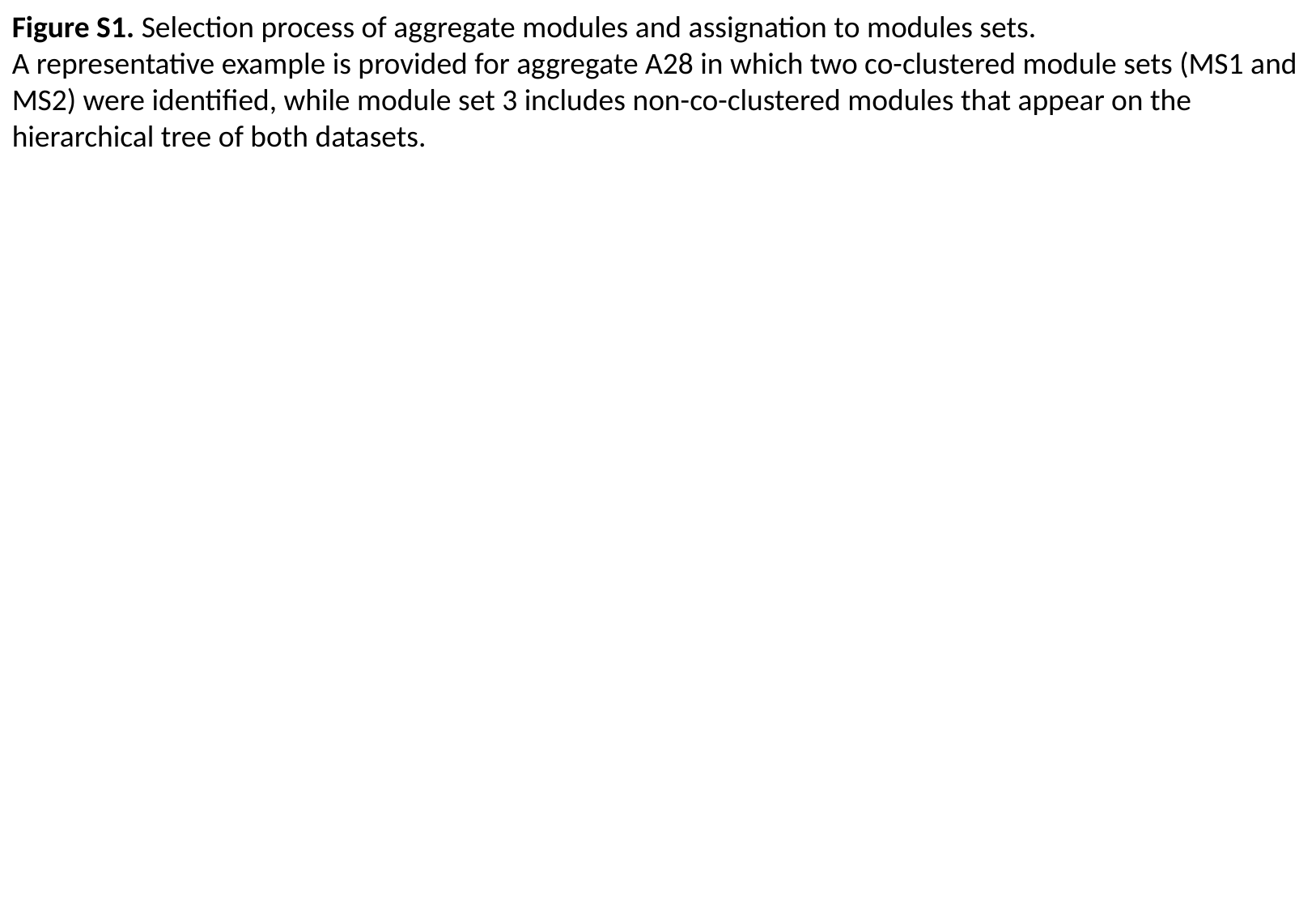

Figure S1. Selection process of aggregate modules and assignation to modules sets.
A representative example is provided for aggregate A28 in which two co-clustered module sets (MS1 and MS2) were identified, while module set 3 includes non-co-clustered modules that appear on the hierarchical tree of both datasets.

## Slide 3
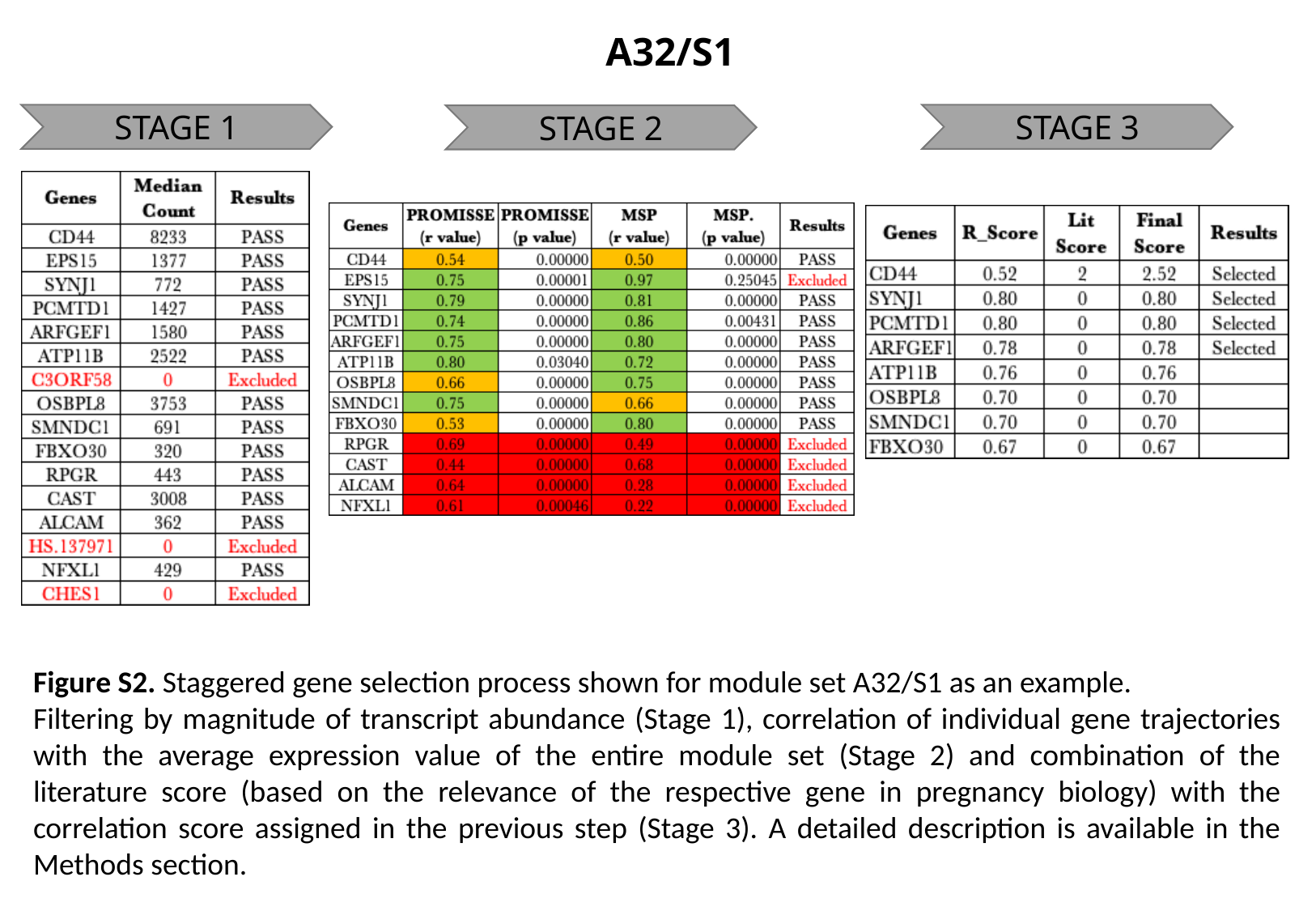

A32/S1
STAGE 1
STAGE 3
STAGE 2
Figure S2. Staggered gene selection process shown for module set A32/S1 as an example.
Filtering by magnitude of transcript abundance (Stage 1), correlation of individual gene trajectories with the average expression value of the entire module set (Stage 2) and combination of the literature score (based on the relevance of the respective gene in pregnancy biology) with the correlation score assigned in the previous step (Stage 3). A detailed description is available in the Methods section.

## Slide 4
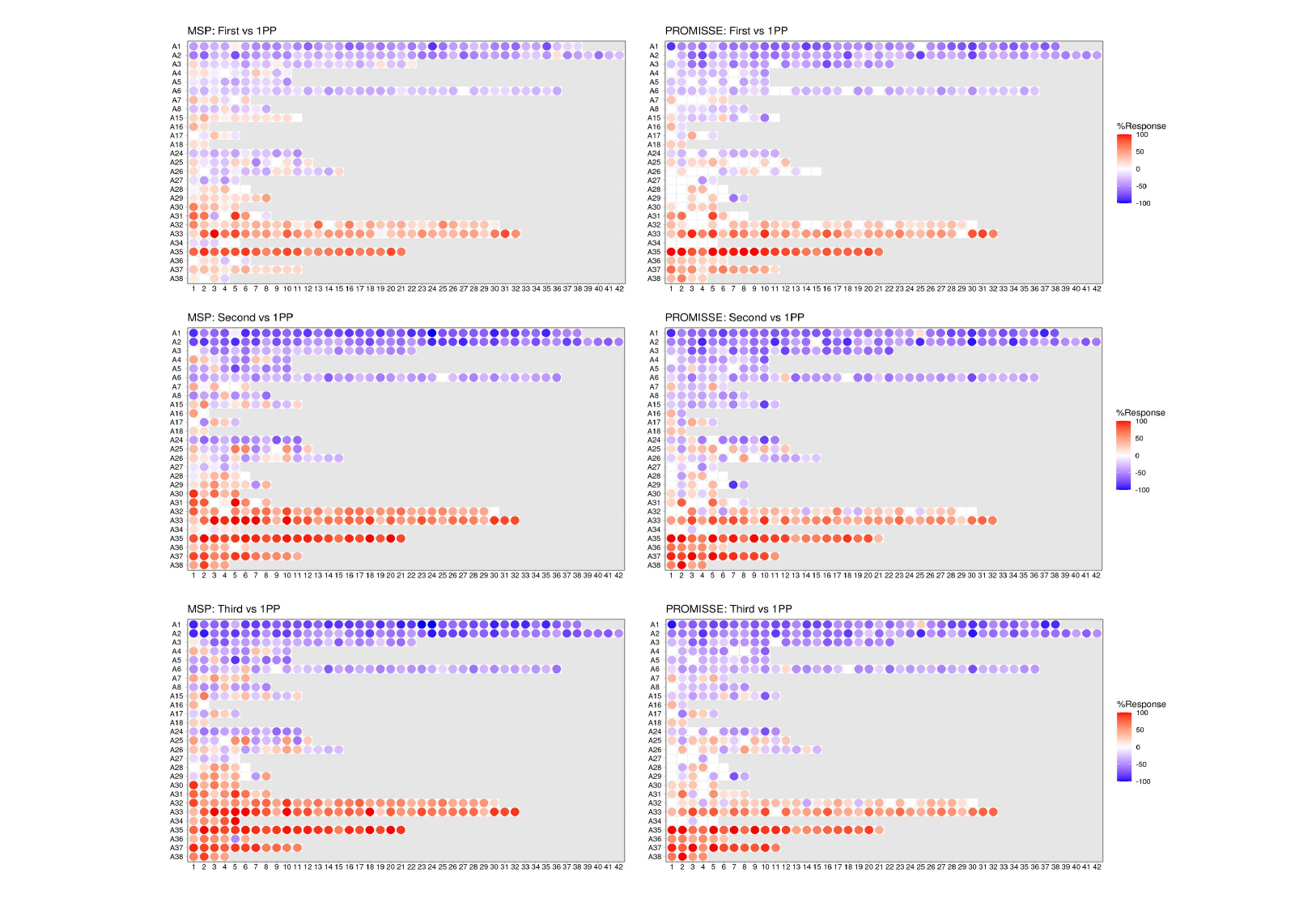

## Slide 5
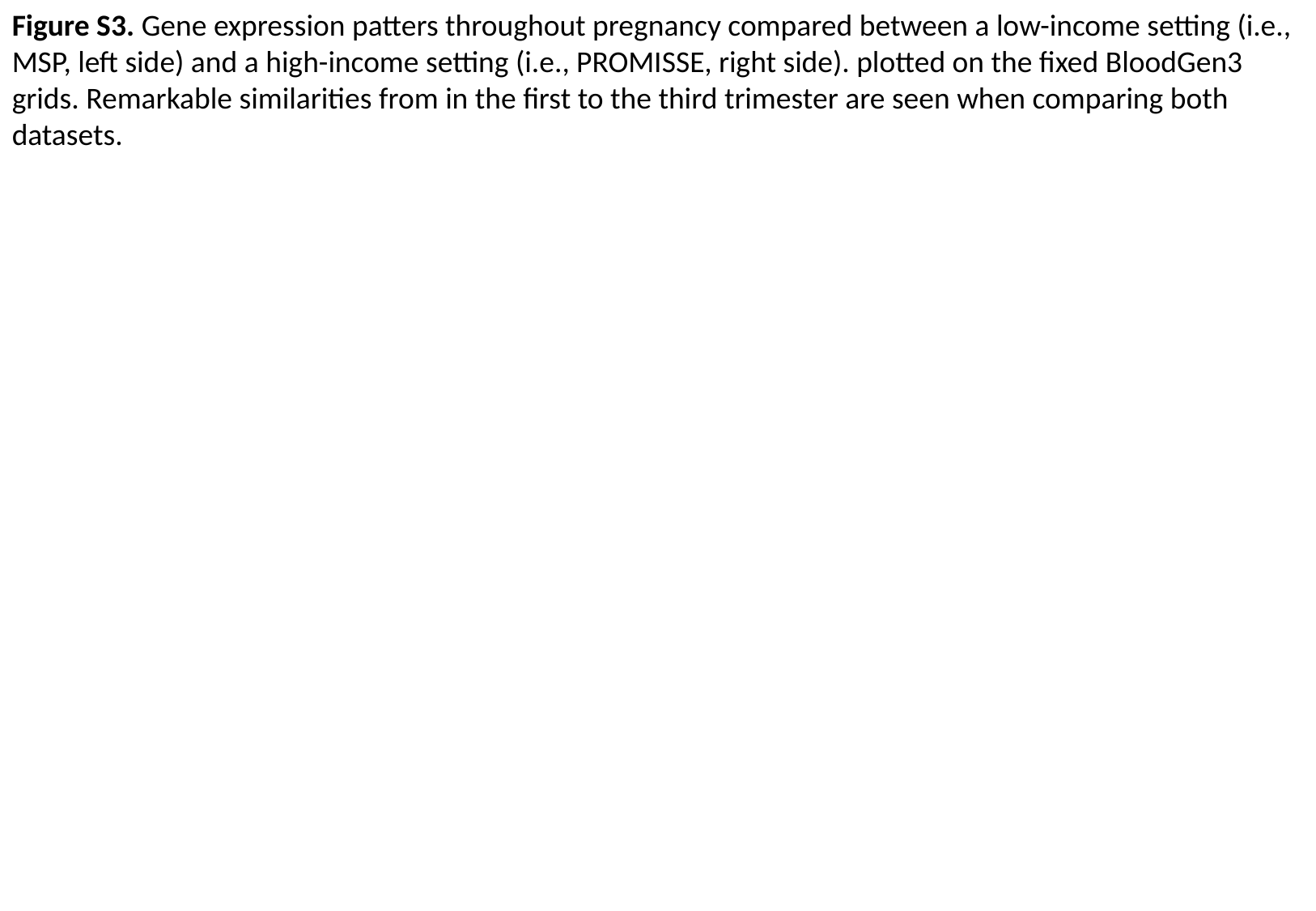

Figure S3. Gene expression patters throughout pregnancy compared between a low-income setting (i.e., MSP, left side) and a high-income setting (i.e., PROMISSE, right side). plotted on the fixed BloodGen3 grids. Remarkable similarities from in the first to the third trimester are seen when comparing both datasets.

## Slide 6
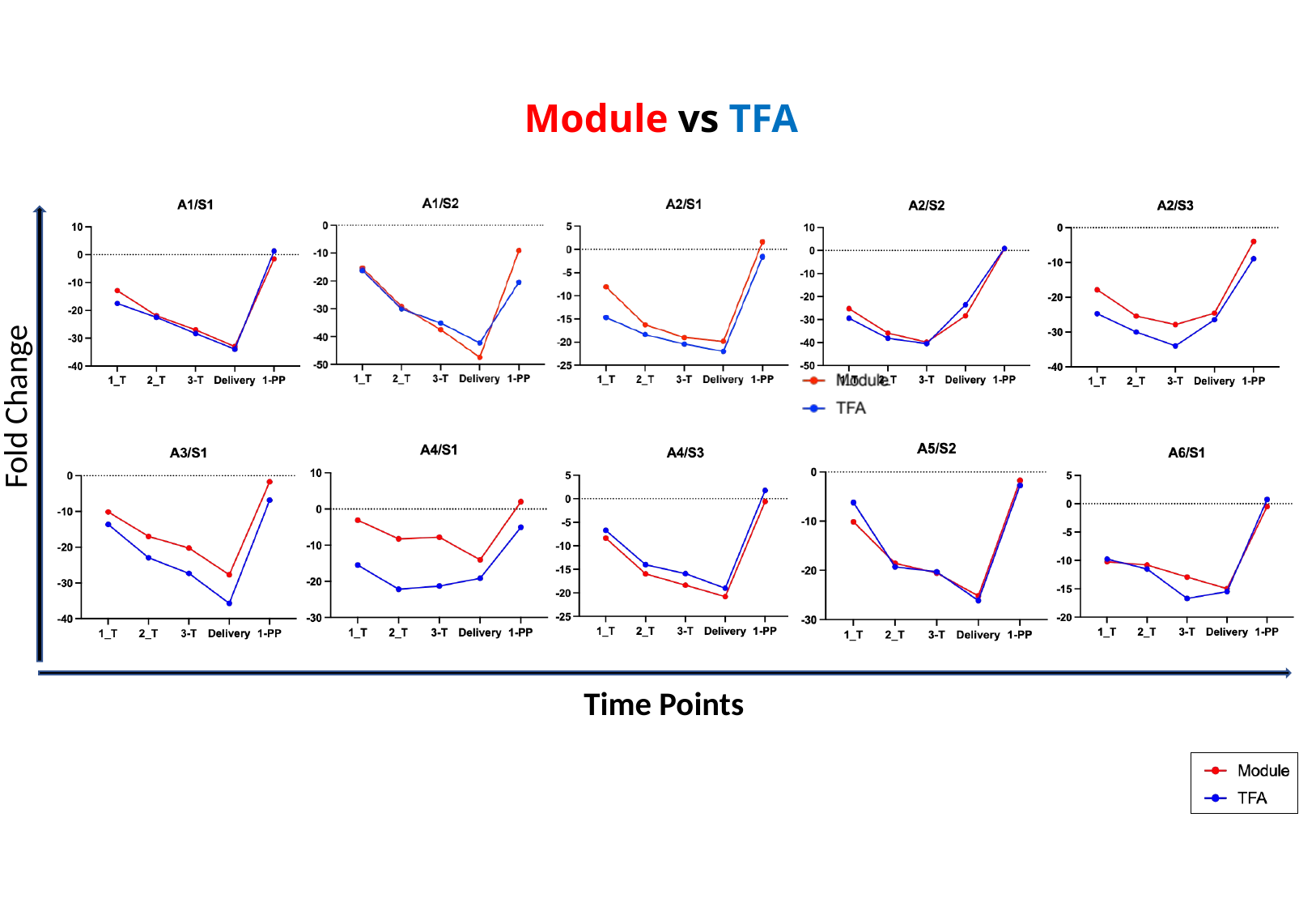

Module vs TFA
Fold Change
Time Points

## Slide 7
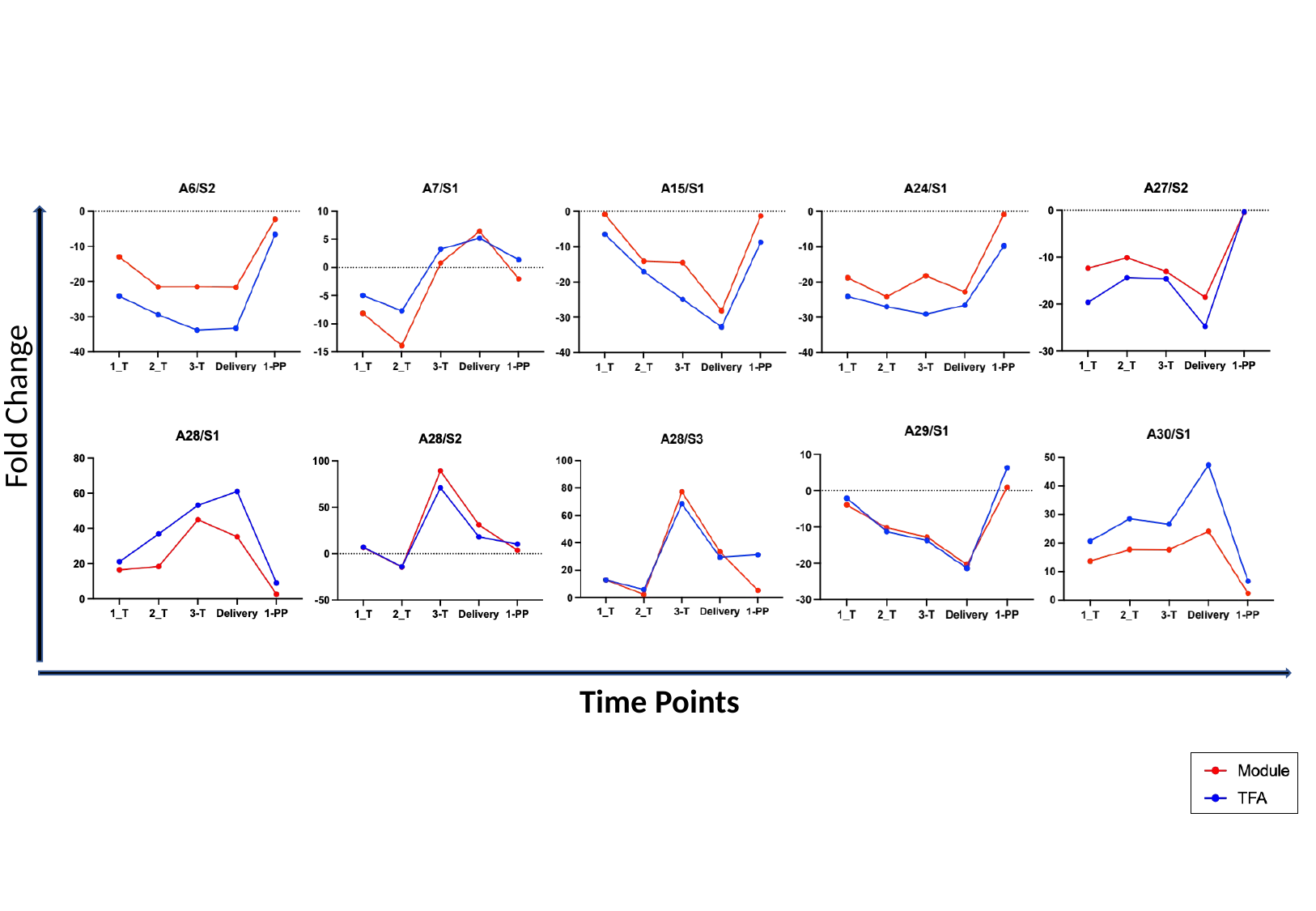

Fold Change
Time Points

## Slide 8
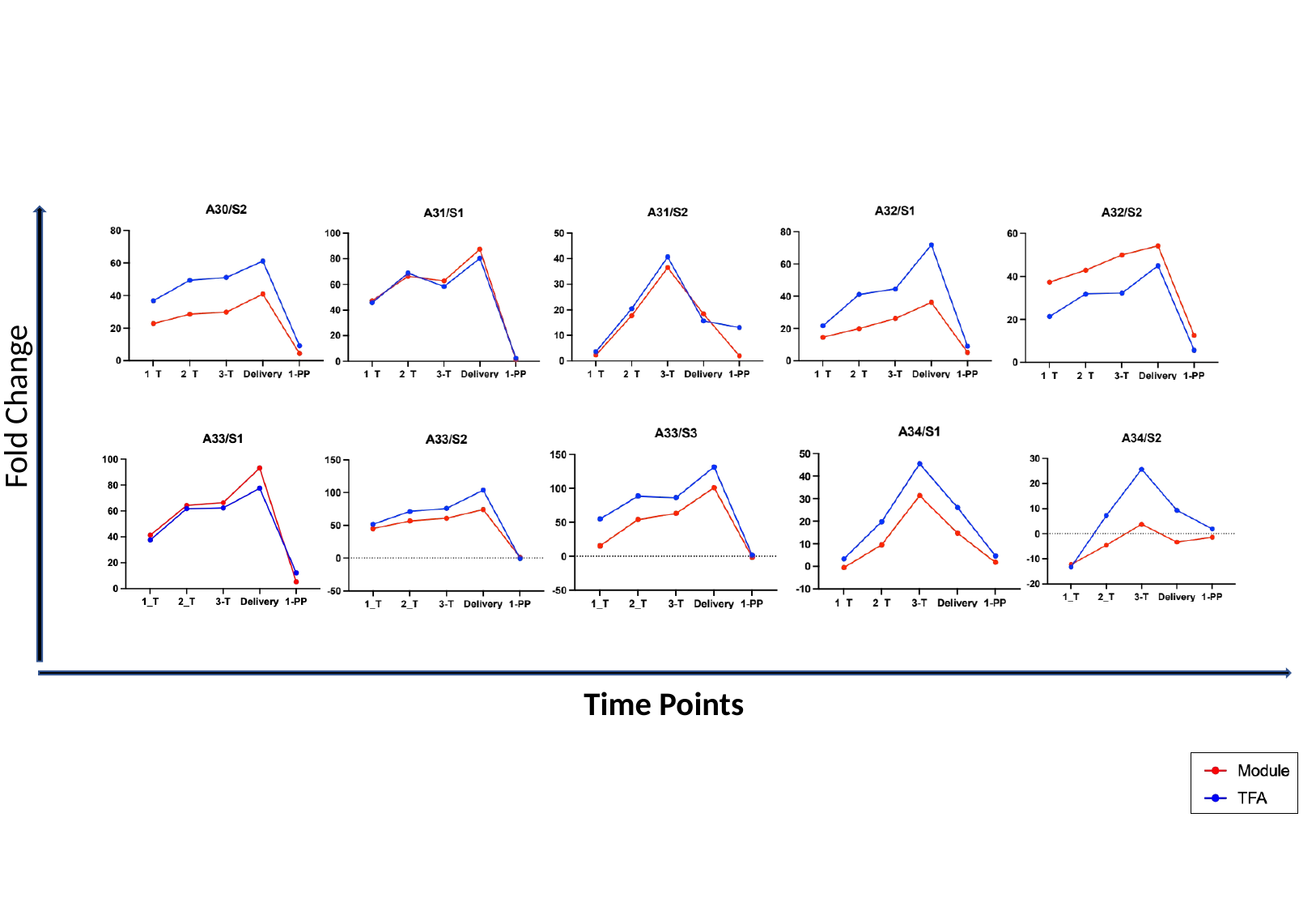

Fold Change
Time Points

## Slide 9
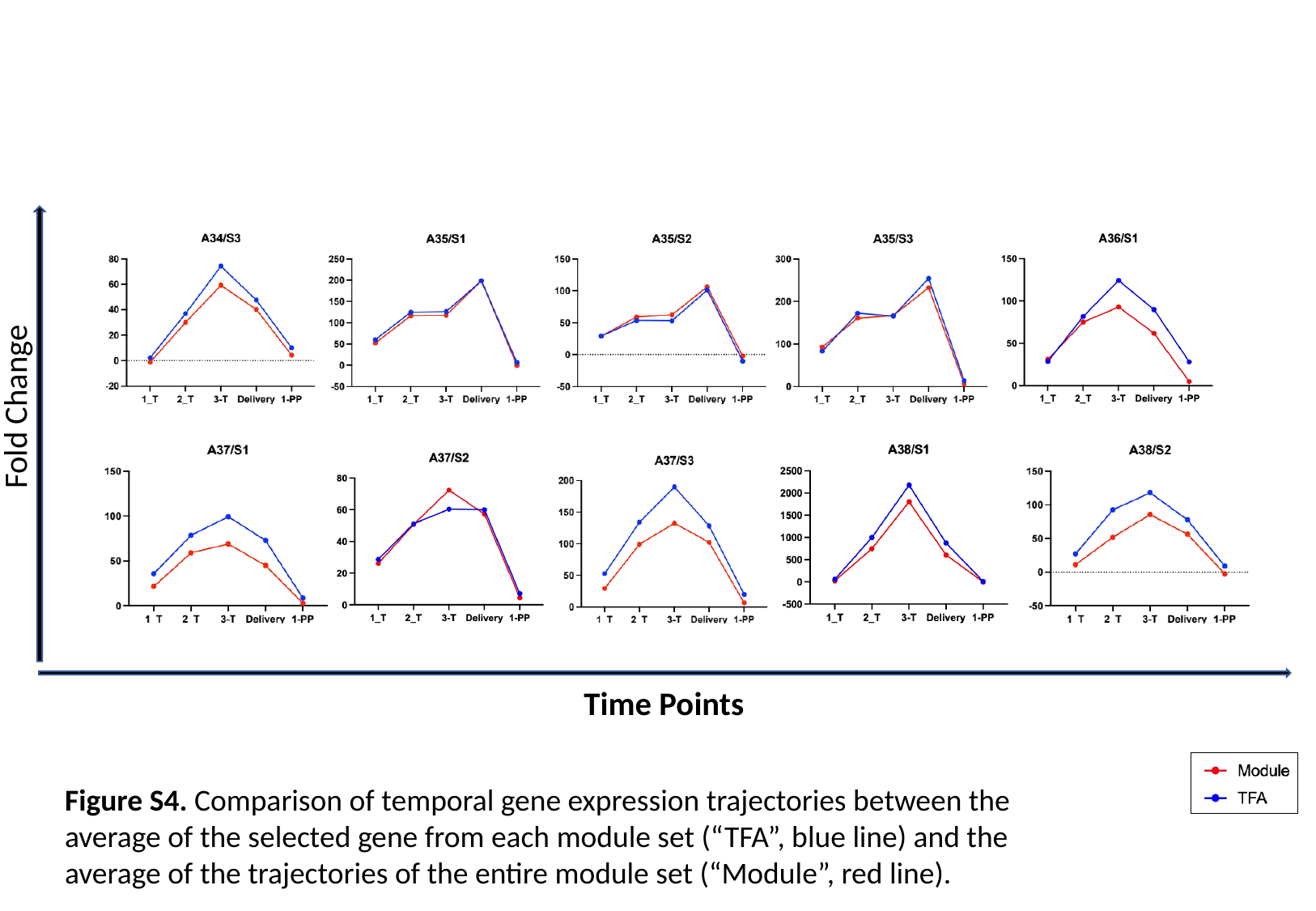

Fold Change
Time Points
Figure S4. Comparison of temporal gene expression trajectories between the average of the selected gene from each module set (“TFA”, blue line) and the average of the trajectories of the entire module set (“Module”, red line).
