# Supplementary material for: Design of a targeted blood transcriptional panel for monitoring immunological changes accompanying pregnancy
Source: Front Immunol. 2024 Jan 30;15:1319949. doi: 10.3389/fimmu.2024.1319949 (PMC10861739; doi:10.3389/fimmu.2024.1319949)
Supplement: Supplementary file 2 [file Table_1.docx]

**Supplementary Table 1: Demographic information of MSP and PROMISSE studies**

| **Demographics** | **MSP Study** | **PROMISSE Study** |
| --- | --- | --- |
| Number of Subjects | 15 | 38 |
| Age (Years, Mean±SD) | 26.4±7.62 | 31.2±4.54 |
| Gravidity (Median (IQR)) | 2 (1,5) | Data not available |
| Parity (%nulliparous) | 33.3% | Data not available |
| BMI at enrollment | 20.56±3.43 | Data not available |
| Gestational age at delivery | 40±0.86 | 39±0.98 |
| Birthweight (kg, Mean±SD) | 3.01±0.32 | Data not available |

**Supplementary Table 2:** Comparison of basic characteristics of the de novo generated dataset (i.e., MSP) and the dataset accessed from the public domain (i.e., PROMISSE).

|  | **MSP Study** | **PROMISSE Study** |
| --- | --- | --- |
| Study population | Uneventful pregnancies | Uneventful pregnancies |
| Study setting | Low-income setting  Thailand-Myanmar Border | High-income setting |
| Subjects | 15 | 38 |
| Sampling timepoints | <14 weeks EGA  21-23 weeks EGA  31-33 weeks EGA  Delivery  1-month postpartum  3-month postpartum | <16 weeks  16-23 weeks  24-31 weeks  32-40 weeks  Postpartum |
| Platform | RNAseq | Illumina BeadArrays |
| Publicly available | Not yet | GSE108497 |

Caption:

Abbreviations: EGA, estimated gestational age; MSP, Molecular Signature in Pregnancy; PROMISSE, Predictors of pRegnancy Outcome: bio-Markers in antiphospholipid antibody syndrome and Systemic Lupus Erythematosus.
